# The role of glucagon after bariatric/metabolic surgery: much more than an “anti-insulin” hormone

**DOI:** 10.3389/fendo.2023.1236103

**Published:** 2023-08-11

**Authors:** Gonzalo-Martín Pérez-Arana, Alfredo Díaz-Gómez, José Bancalero-de los Reyes, Manuel Gracia-Romero, Antonio Ribelles-García, Francisco Visiedo, Álvaro González-Domínguez, David Almorza-Gomar, José-Arturo Prada-Oliveira

**Affiliations:** ^1^ Department of Human Anatomy and Embryology, University of Cadiz, Cádiz, Spain; ^2^ Institute for Biomedical Science Research and Innovation (INIBICA), University of Cadiz, Cádiz, Spain; ^3^ San Carlos Hospital, Andalusian Health System, Cádiz, Spain; ^4^ Complejo Hospitalario de Badajoz, Servicio Extremeño de Salud, Cádiz, Spain; ^5^ Operative Statistic and Research Department, University of Cádiz, Cádiz, Spain

**Keywords:** glucagon, diabetes, sleeve gastrectomy, Roux-en-Y gastric bypass, incretin, glucose, alpha cell

## Abstract

The biological activity of glucagon has recently been proposed to both stimulate hepatic glucose production and also include a paradoxical insulinotropic effect, which could suggest a new role of glucagon in the pathophysiology type 2 diabetes mellitus (T2DM). An insulinotropic role of glucagon has been observed after bariatric/metabolic surgery that is mediated through the GLP-1 receptor on pancreatic beta cells. This effect appears to be modulated by other members of the proglucagon family, playing a key role in the beneficial effects and complications of bariatric/metabolic surgery. Glucagon serves a dual role after sleeve gastrectomy (SG) and Roux-en-Y gastric bypass (RYGB). In addition to maintaining blood glucose levels, glucagon exhibits an insulinotropic effect, suggesting that glucagon has a more complex function than simply an “anti-insulin hormone”.

## 1 Introduction

Glucagon, which was first identified in 1922, is a 29-amino acid peptide hormone that is produced and secreted by the alpha cell population within the pancreatic islets (1). In conjunction with insulin, glucagon serves to maintain glucose homeostasis. Glucagon stimulates hepatic glycogen breakdown via glycogen phosphorylase and gluconeogenesis via fructose 1,6-bisphosphatase activation; both mechanisms lead to elevated blood glucose (2). Glucagon also acts on lipid metabolism, stimulating beta-oxidation of fatty acids and facilitating the transport of fatty acids to the mitochondria by increasing the activity of carnitine acyltransferase (3). Diabetic hyperglycemia is often described as the result of impaired insulin secretion, but diabetic hyperglycemia also occurs due to inadequate glucagon release. This relationship has been reinforced by recent findings that glucagon/GLP-1 receptor dual agonists lead to improved blood glucose in type 2 diabetes mellitus (T2DM) patients.

Bariatric surgery (BS)/metabolic surgery (MS) has become the most powerful tool against T2DM (4). The study of the mechanisms by which BS/MS improves T2DM has included the investigation of a group of peptides called incretins. Among them are glucagon-like peptide-1 (GLP-1) and glucose-dependent insulinotropic polypeptide (GIP), as well as other peptides, which although we cannot consider incretins, are related such as peptide tyrosine-tyrosine (PYY) and ghrelin. The effects of incretins on the production, secretion, and action of insulin have been widely studied in animal models and humans. We focus on their effects on glucagon secretion and actions related to the two most performed variants of this surgery, sleeve gastrectomy (SG) and Roux-en-Y gastric bypass (RYGB).

## 2 Methods

**Figure 1:**
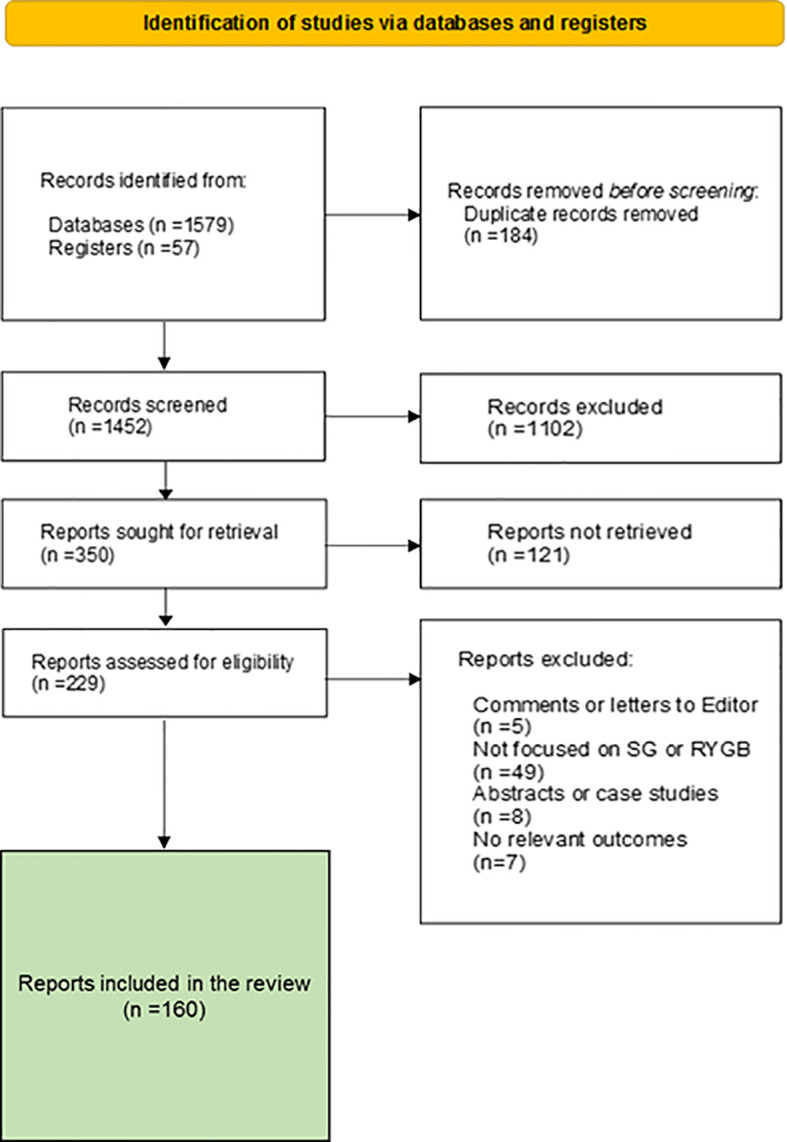
PRISMA flow diagram of the study.

To achieve our goal, we performed a selective search of scientific articles in many databases according to the PRISMA protocol (**Figure 1**). The protocol and results were registered at the RODIN-University of Cádiz repository (http://hdl.handle.net/10498/27147). The search was limited to standard scientific databases. The Boolean operators used were AND, OR and NOT, and the key words were Glucagon, GLP-1, Incretin, Sleeve Gastrectomy, Roux-en-Y Gastric Bypass, Glucose metabolism, Glycolysis and alpha cell. A total of 160 reports were selected for the preparation of this manuscript. We reduced the number to 50 main references according to editorial determination.

## 3 Glucagon secretion and modulating agents: the role of incretins

Preproglucagon is encoded by the Gcg gene, which is expressed in pancreatic alpha cells, L cells from the gut and many neurons from the nucleus of the solitary tract in the brain (5). Different products are produced in these cell types depending on preproglucagon processing by different prohormone convertase family members (PCs) (6). PC2 is exclusively responsible for the production of glucagon in pancreatic alpha cells, whereas PC1/3 is responsible for the formation of glucagon-like peptide-1 (GLP-1) in the same cells (7). In addition, PC1/3 is present in other cell types, such as neurons and L-cells producing GLP-1, GLP-2, glicentin and oxyntomodulin (8).

Glucagon is primarily produced in alpha cells and its secretion is stimulated by low blood glucose levels. When low blood glucose levels are detected, circulating glucose is taken up by alpha cells via glucose transporter 1 (GLUT1), which exhibits high glucose affinity. The low intracellular ATP/ADP ratio in the alpha cell leads to ATP-sensitive potassium (KATP) channel closure. Depolarization of the plasma membrane and, ultimately, voltage-dependent calcium channel activation allow the flow of calcium into cells and glucagon exocytosis mediated by the SNARE complex of exocytosis proteins (9).

However, low glucose is not the only important stimulus for glucagon release. Amino acids (AAs) have long been known to stimulate glucagon release. The most powerful stimulators of glucagon release include arginine, alanine and glutamine. Recently, glutamine-stimulated glucagon secretion has been linked to high interstitial glutamate concentrations and glutamate-induced changes in plasma membrane polarization (7). Thus, the presence of high plasma levels of AAs induces a strong glucagonotropic effect, preventing potential hypoglycemia due to AA-induced insulin secretion (8). Moreover, high glucagon plasma levels induce hepatic uptake of AAs and activation of the urea cycle in the liver, leading to enhanced hepatic AA turnover and hypoaminoacidemia. Thus, a feedback relationship exists between the liver and the alpha cell population (10).

Glucagon secretion can be modulated by a number of factors, including insulin secretion itself or the release of somatostatin by pancreatic delta cells, limiting glucagon and insulin release (11) (**Table 1**). In this context, free fatty acids (FFAs), among many other factors, seem to play a key role in the upregulation of glucagon and insulin secretion through FFAR4/GPR120 receptor binding in delta cells and decreasing somatostatin release by pancreatic delta cells. Additionally, infusion of amylin, a 37-amino-acid peptide produced by beta cells at a ratio of 1:100 to insulin, limits arginine-stimulated glucagon secretion in rats (12). Pancreatic polypeptide (PP), a member of the neuropeptide Y family released by PP cells within the islets, acts on alpha and delta cells to inhibit glucagon and somatostatin secretion as well as binding to PPYR1, the Y4 subtype of the G-protein coupled receptor (GPCR) family.

**Table 1:** Effects of gastrointestinal hormones on glucagon secretion.

Hormone	Effect on glucagon secretion	Mechanism of action
Gastrin	Unknown	----------
Glucagon-Like Peptide 1 (GLP-1)	Negative	Indirect (SST Mediated)
Glucagon-Like Peptide 2 (GLP-2)	Positive	Direct
Glucose-dependent Insulinotropic Peptide (GIP)	Positive	Direct
Ghrelin	Positive	Direct
Insulin	Negative	Direct
Secretin	Unknown	Direct
Somatostatin (SST)	Negative	Direct
Oxyntomodulin (OXM)	Positive	Direct
Peptide Tyrosine-Tyrosine (PYY)	Unknown	----------

### 3.1 GLP-1

Among the glucagon-secretion modulators, a few gastrointestinal hormones deserve special mention. The first is GLP-1, a peptide of its own family primarily produced in intestinal L cells. A very small portion of GLP-1 is also produced and locally released within the pancreatic islets by alpha cells (13). The effect of GLP-1 on glucagon secretion is controversial; the presence of GLP-1 on isolated alpha cell cultures leads to enhanced glucagon secretion; however, many authors have proposed an inhibitory effect in isolated pancreas and GLP-1-infused patients. This finding suggests an indirect mechanism mediated by an intermediate factor. Supporting relationship, the GLP-1 receptor (GLP-1R) has been detected on the surface of pancreatic delta cells, and perfusion of somatostatin-14 (SST-14) in isolated rat pancreas led to diminished glucagon release by alpha cells (14).

### 3.2 GIP

GIP is another gastrointestinal incretin hormone produced by K cells, primarily in the duodenum. GIP is able to enhance glucagon secretion of alpha cells in rats, healthy people or patients with T2DM both in the hyperglycemic and euglycemic state. This effect appears to be direct and due to the binding of GIP to its receptor (GIPR) on the alpha cell surface (15) and resulting increases in intracellular calcium concentrations, leading to glucagon vesicle release (16). Some authors propose that loss of beta cell GIPR function due to cell stress in T2DM patients may reduce the inhibitory effect of insulin on glucagon secretion, leading to elevated GIP-stimulated glucagon secretion even during hyperglycemia (17), but this hypothesis is controversial.

### 3.3 Ghrelin

The gastrointestinal peptides that act on glucagon secretion also include ghrelin, a 28-amino-acid peptide primarily produced by X/A-like cells in the oxyntic glands of the gastric fundus. A small amount of ghrelin is also produced in the pancreatic islets by epsilon cells (18). Ghrelin receptor (GHSR) has been detected in alpha cell populations, and a direct stimulating effect of acyl ghrelin on glucagon secretion was observed in a pancreatic alpha cell line clone in isolated islets (19). The exact mechanism of ghrelin-enhanced glucagon secretion remains unknown.

### 3.4 GLP-2

GLP-2 is another enterohormone encoded by the Gcg gene and processed by PC1/3 in intestinal L cells. GLP-2 has also been described inside the pancreatic islets, and a glucagonotropic effect occurs after intravenous administration of GLP-2 to patients (20). Thus, GLP-2 may act directly by binding to its receptors on alpha cells, leading to glucagon release. However, cross-activation of GLP-1R by other members of the preproglucagon family has also been described. Therefore, we cannot rule out activation of GLP-2R on the surface of alpha cells by GLP-1 or by the released glucagon, thereby serving as a mechanism to amplify glucagon secretion.

### 3.5 Oxyntomodulin

Another interesting peptide is oxyntomodulin (OXM), a peptide related to GLP-1 and GLP-2 obtained from proglucagon processing by PC1/3 in L cells along the intestine but also at the caudal part of the nucleus of the solitary tract in the Central Nervous System (CNS) (21). OXM stimulates glucagon secretion by binding to glucagon receptors (GCGR) with low affinity, as has been demonstrated *in vitro* and in animal models (22). In practice, OXM is rapidly degraded by the DPP-4 enzyme, leading to a small amount of circulating OXM and negligible activity in carbohydrate metabolism.

### 3.6 PYY

PYY is a molecule co-secreted with GLP-1 by intestinal L cells. Its mechanism of action on alpha cells remains unknown, but PYY has been shown to stimulate glucagon production in cultured islets from diabetic GK rats. More recently, PYY expression inside pancreatic islets from rodents and humans and GLP-1–mediated paracrine activity in islets have been documented. However, the sensitivity of PYY to degradation by DPP-4 activity and the limited quantities in which it is expressed makes studying PYY difficult (23).

## 4 Glucagon and glucose metabolism

### 4.1 Physiological effects of glucagon on glucose metabolism

The regulation exerted by the insulin–glucagon pair on carbohydrate metabolism is well known. After a meal, high blood glucose levels stimulate insulin release by pancreatic beta cells, leading to glucose uptake and glycogen production in the liver and muscles in a process called glycogenesis, and insulin also inhibits glucagon release.

Glucagon is secreted by the pancreatic alpha cell population in situations of hypoglycemia or stress, reaching blood concentrations of up to 1 ng/mL in such situations. When glucagon reaches the target tissues, it binds to glucagon receptor (GCGR), a G-protein-coupled receptor with seven transmembrane domains on the cell surface.

The effects of glucagon in the liver also suggest paradoxical situations, such as those related to protein synthesis in fasting periods through transient protein kinase mechanistic target of rapamycin 1/2 (mTORC1/2) activation thanks to the GTPase activity of RAP-1 aided by exchange protein activated by cAMP (EPAC). The increase in hepatic sensitivity to insulin induced by glucagon due to phosphorylation of AKT at Ser 473 is similar to the effect of insulin on mTORC2, which has been observed in primary cultures of hepatocytes and is of special therapeutic interest (24).

**Figure 2:**
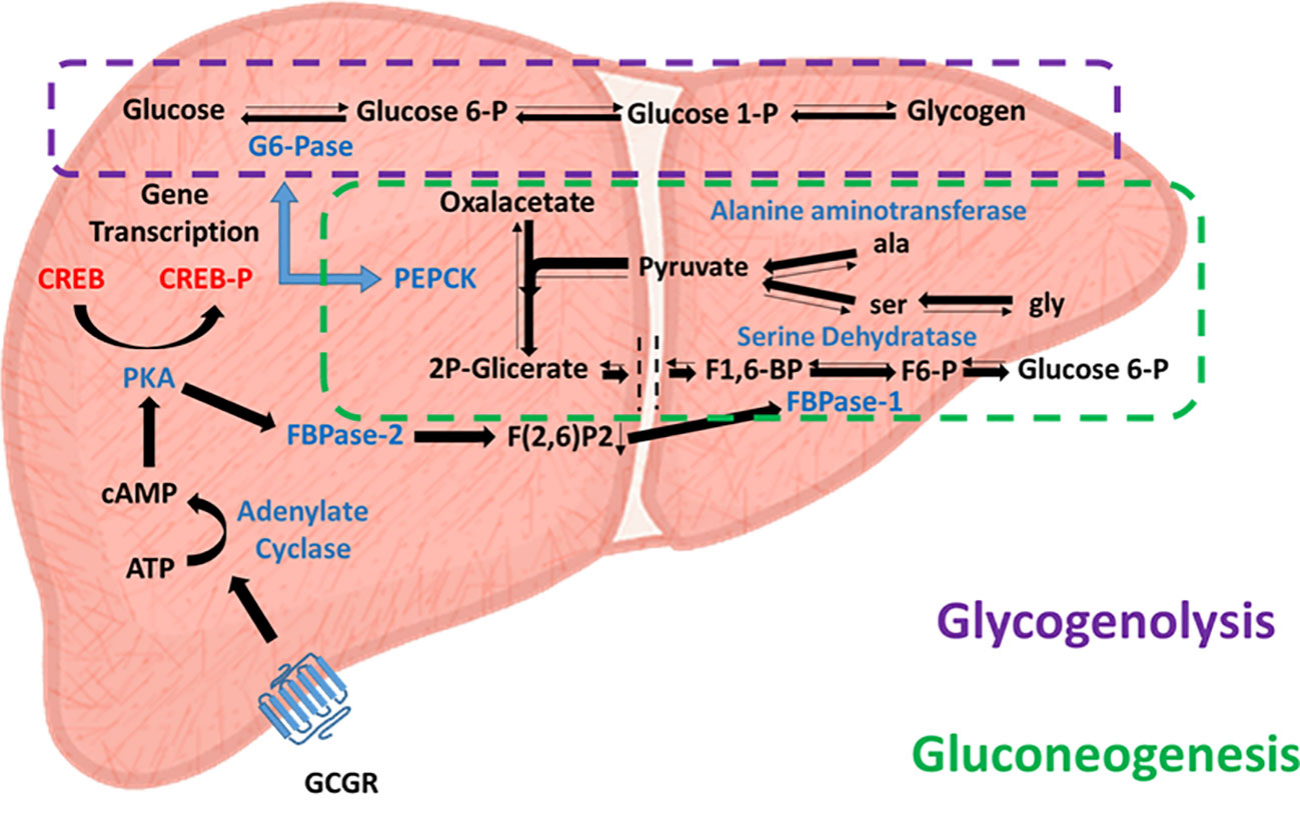
Glucagon-activated glycogenolytic and gluconeogenic pathways. Effects of glucagon receptor (Cgcr) activation on hepatic glycolysis (dashed purple line) and gluconeogenesis (dashed green line) pathways. Enzymes involved are in blue, substrates are in black, and transcription factors are in red.

After a meal, high blood glucose levels stimulate insulin release from the pancreatic beta cell population to the bloodstream, inhibit the pancreatic secretion of glucagon and enhance glucose uptake and glycogen storage, primarily in the liver and muscles. These effects lead to decreased blood glucose levels, facilitating euglycemia. In contrast, during fasting periods of 2 to 12 hours, blood glucose level depletion is prevented by the action of glucagon secreted by the pancreatic alpha cell population. As mentioned above, this initially induces glycogenolysis in peripheral tissues and the liver, but when glycogen reserves are depleted, glucagon also induces hepatic gluconeogenesis, ensuring maintenance of proper blood glucose levels. However, the role of glucagon in hepatic gluconeogenesis is complex; a recent study proposed that the mechanism of gluconeogenesis is more dependent on the availability of gluconeogenic amino acids than on the action of glucagon itself (24). Glucagon increases the availability of gluconeogenic amino acids. In this sense, glucagon regulates hepatic glucose metabolism more by the glycogenolysis pathway than by gluconeogenesis. This relationship is supported by the poor hepatic glucose production after glucagon administration in patients with low hepatic glycogen reserves subjected to a low carbohydrate diet. Therefore, glucagon may lead to immediate regulation mediated by glycogenolysis, or medium-term regulation mediated by gluconeogenesis (**Figure 2**).

### 4.2 Glucagon and type 2 diabetes mellitus

Patients with type 2 diabetes mellitus (T2DM) exhibit high plasma glucagon levels in the fasting periods and lack the ability to inhibit its secretion after a meal. Historically, steps have been taken to ameliorate the disease. Diabetic patients were administered somatostatin, which limited hyperglycemia in most patients (25). Later, glucagon receptor (Gcgr -/-) knockout mice treated with streptozotocin also exhibited euglycemia without hypoglycemic episodes in an interesting study (26). However, in this model, worse glycemic control may develop if GCGR ablation is induced after the onset of STZ-induced diabetes. Moreover, this partial control of hyperglycemia was lost when the animals were treated with a GLP-1R antagonist. These findings lead us to consider the establishment of an alternative pathway to GCGR after its ablation, the role of GLP-1R in this alternative pathway and the need for time to establish the pathway. Since that time, several studies have tested the GCGR antagonist effect on glucose metabolism in animals and humans with T2DM, confirming beneficial antidiabetogenic effects (27). However, the use of GCGR antagonists as pharmaceuticals has drawbacks, such as a notable increase in hepatic transaminases and LDL cholesterol (28). Other ways of limiting the effect of glucagon on glucose metabolism that have been studied include the use of anti-GCGR antibodies, which is efficient but also exhibited many problems in preclinical trials, such as alpha cell hyperplasia in treated animals, a common problem for any agents that disrupts glucagon receptor signaling (29). Similar beneficial effects on glucose metabolism have been demonstrated by Gcgr-antisense oligonucleotide administration, which leads to inhibited Gcgr expression; only mild and local side effects have been observed when these oligonucleotides are used (30).

Paradoxically, glucagon stimulates dose-dependent insulin release via GLP-1R in beta cells in rodents (31). Considering the therapeutic role of GLP-1R agonists such as exenatide, liraglutide, or lixisenatide on glucose tolerance (32), it is easy to understand why the combined effect of glucagon/GLP-1 dual co-agonists on glucose and lipid metabolism is being widely studied (33) and why they might exhibit a superior and more complete effect than single agonists. Therefore, several glucagon/GLP-1R co-agonists have been developed with different affinities for the two receptors and varying effects on glucose tolerance in rats and mice, likely due to different glucagon receptor tissue distributions in each receptor. Despite this difficulty, a handful of these co-agonists, as LY2944876/TT-401, LY3305677 (Eli Lilly), SAR425899 (Sanofi-Aventis), Efinopegdutide (Merk), BI 456906 (Boehringer Ingelheim), Efocipegtrutide (Hanmi Pharmaceutical), JNJ-54728518 (Jannsen) or cotadutide (AstraZeneca), have moved to different phases of clinical trials (33–36) oriented toward the treatment of obesity rather than diabetes. Finally, a triple agonist of GIPR, GLP1R and GCGR called Retratutide has also recently been tested in a phase 2 trial. This showed a great ability to substantially reduce body weight. However, it also presented a series of adverse gastrointestinal events, although limited to patients treated with high doses (37).

## 5 Glucagon and metabolic surgery

**Figure 3:**
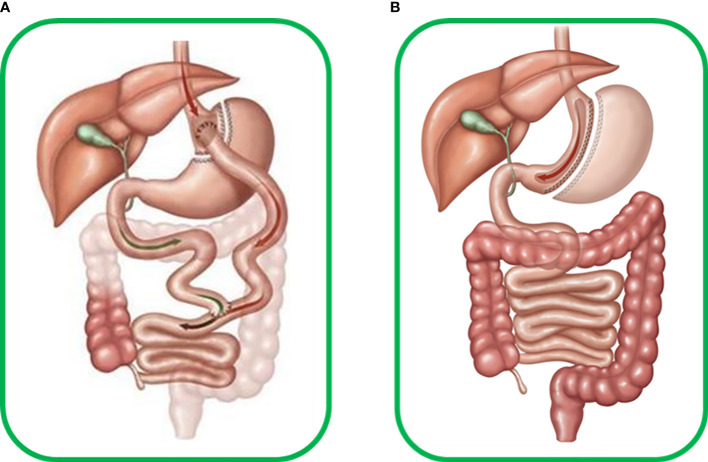
**(A)** Human Roux-en-Y gastric bypass surgery (RYGB) includes transverse section of the stomach from the great to the minor curvature, producing a new gastric pouch with limited volume that continues to the alimentary limb connecting to the medium portion of the jejunum. The antrum, duodenum, and initial portion of jejunum form the biliopancreatic limb, which excludes food flux. Thus RYGB becomes a mixed malabsorptive and restrictive technique. **(B)** Human sleeve gastrectomy (SG) surgery, which involves important reduction of around 80-95% of total gastric volume due to resection of most of great gastric curvature with ablation of most of the antrum, stomach corpus, and gastric fundus, forming a cylindrical pouch and preserving the pylorus and minor gastric curvature, thus becoming a restrictive technique.

Today, metabolic surgery is one of the most effective weapons in the therapeutic arsenal against T2DM, and since its appearance, many surgical variants have been developed with varying degrees of success (4). Two approaches stand out for their safety and efficiency: Roux-en-y-gastric bypass (RYGB) and Sleeve gastrectomy (SG). RYGB is a surgery involving mixed malabsorptive and restrictive components that limit the volume of the stomach and the absorption length of the small intestine, whereas SG is a restrictive surgery that only affects the gastric volume (**Figure 3**). Most studies on the pathophysiological mechanisms underlying the improvement of glucose metabolism after these surgeries focus on insulin production and secretion (38), and few focus on the effect on glucagon, often resulting in an incomplete view of the phenomena that lead to changes in glucose metabolism after surgery. We have compiled and summarized what is known about the role of glucagon after these two surgeries.

### 5.1 The role of glucagon after RYGB

The induction of postprandial hyperglucagonemia in patients undergoing RYGB has long been observed (39). However, other bariatric techniques that involve duodenal exclusion, such as the duodenal-endoluminal sleeve (DES), seem to stimulate hyperglucagonemia (40). This is a paradoxical situation given that RYGB also stimulates insulin secretion and limits high glucose levels. However, supporting these observations, long-term increased alpha cell mass has been observed in Wistar rodent models after RYGB (41), and elevated plasma glucagon levels have been observed after the oral glucose tolerance test (OGTT) but not after intravenous glucose administration in T2DM patients after RYGB (39). Furthermore, glucagon gene expression and glucagon expression are increased in patients after RYGB, as determined by small intestine biopsy, indicating new sources of alternative glucagon secretion. This finding may be explained by modification of the intestinal mucosa and glucagon gene processing by L cells due to early contact with food derived from RYGB surgery. However, the number of patients studied is small, and recent studies combining liquid chromatography and mass spectrometry techniques did not observe increased fasting or postprandial glucagon levels in T2DM patients one year after RYGB (42). These findings therefore raise suspicions of cross-detection of other proglucagon-like molecules, such as glicentin or OXM (43). It is necessary to establish the sensitivity and specificity of these techniques and determine which is the most suitable for this type of study. In this sense, a recent study has established a close correlation between glucagon levels measured using sandwich enzyme-linked immunosorbent assay (ELISA) in pancreatectomized patients and glucagon-related hormone levels such as OXM or glicentin. These findings lead us to consider a cross-reaction between molecules of the same family. Therefore, it is necessary to confirm the hyperglucagonemia detected after RYGB using other techniques, preferably LC-MS. Meanwhile, all data referring to the presence and amount of glucagon announced in the studies should be taken with great caution.

The important role that glucagon could play after RYGB is also not clear. Episodes of severe postprandial hypoglycemia are one of the most important and frequent complications following RYGB surgery. Some authors attribute this phenomenon to an excessive and inappropriate insulin response after the meal. However, the published data are inconclusive. . In addition, there is a C-terminal 19-29 mini-glucagon from the fragmentation of glucagon during its processing and released together with it. Mini-glucagon 19-29 has a potent limiting effect on insulin secretion (44, 45) and it could happen that RYGB modifies the released ratios of glucagon/mini-glucagon 19-29, which could explain the episodes of postprandial hypoglycemia.

At the same time, many authors have proposed that the high postprandial plasma glucagon level is the key to avoiding severe hypoglycemic episodes after meals in RYGB patients (46). However, Tharakan et al. proposed that the combination of early elevated plasma glucagon and GLP-1 levels after meals may imply an insulinotropic effect triggering postprandial hypoglycemia (47), building on previous studies in which these hypoglycemic states were limited using GLP-1R antagonists. Therefore, some GLP-1R antagonists, such as exendin (9–39), may be of special therapeutic interest. Thus, postprandial glucagon has a dual role. It acts first as a GLP-1 agonist, leading to an insulinotropic effect through GLP-1R, and subsequently stimulates the liver to avoid hypoglycemia as a safety mechanism. In this sense, some experiments performed in diabetic rodents suggest that the response to GLP-1R agonists prior to surgery may act as a predictor of the future effect of RYGB on glucose metabolism and are of significant therapeutic interest. Thus, the response to GLP-1 R prior to surgery would indicate the degree of sensitivity of the patient´s GLP-1R to GLP-1 and other similar molecules such as glucagon. Giving us an idea about the efficiency of surgery to improve glucose metabolism but also about the possibilities of postprandial hypoglycemia episodes after surgery (48).

Another possible explanation for postprandial hypoglycemia is glucagon resistance. Hepatic steatosis and certain diets have been related to decreased hepatic GCGR expression in animals, probably due to GCGR internalization (49). However, given the presence of liver fat in animals and the consumption of a high-fat diet by patients after RYGB, this situation does not persist and postprandial hypoglycemia can be seen even years after bariatric/metabolic surgery. RYGB also appears to increase circulating levels of GIP in diabetic-GK rats. This is of great interest considering the possible synergistic insulinotropic effects established with GLP-1 in cultured diabetic and nondiabetic human islets (50) and its demonstrated insulinotropic effect in T2DM obese patients. However, the role of GIP on glucagon secretion is not entirely clear. Many authors propose a GIP-stimulated glucagon secretion even during hyperglycemia, but this hypothesis is controversial since in Type 1 diabetes patients without insulin secretion, GIP infusion cannot stimulate glucagon secretion during hyperglycemia (51). Therefore, more research is needed in this field to clarify these events.

Previous studies by our group have demonstrated the fundamental role of PYY as a regulatory molecule for the increased secretion of both GLP-1 and GIP after RYGB in diabetic-GK rats when PYY exhibits secretion patterns prior to those of GLP-1 and GIP after ingestion (52).

Within the glucagon family of molecules, GLP-2 also appears to be increased after RYGB (53), which is consistent with high postprandial glucagon levels given the glucagonotropic effect of GLP-2. This relationship suggests a dual role for GLP-2 in maintaining glucose homeostasis indirectly by stimulating glucagon secretion and facilitating the growth and adaptive response of the intestinal absorptive loop (54).

### 5.2 The role of glucagon after SG

Today, SG is the most common restrictive surgical procedure performed in the USA, with undeniable postsurgical benefits for glucose metabolism. Many studies have been conducted to elucidate how SG improves T2DM, implying modified bile acid circulation, elevated amino acid levels, and modified incretin expression (55). We will focus on those that relate changes in glucose metabolism after surgery with glucagon.

Elevated glucagon: insulin ratios have been reported in diabetic-GK rats after SG, and an early increase in serum glucagon levels has been reported in patients. These findings are consistent with what was observed in previous studies by our group reporting a long-term increase in pancreatic alpha cell mass in healthy rodents after SG with potent glucagon secretion capable of buffering exogenous insulin administration (56). This finding could explain the improved insulin secretion reported after SG because beta cell-expressing GCGR is activated by glucagon in rodents. However, beta cell-specific Gcgr knockout animals exhibit normoglycemia, suggesting that the insulinotropic activity of glucagon is mediated mainly through GLP-1R present in beta cell populations (57). Therefore, the elevated plasma GLP-1 levels observed after SG suggest a joint effect of both on insulin release after SG. However, T2DM patients’ serum glucagon levels decrease approximately 6 months after surgery, but the improved glucose metabolism persists (58). Could this phenomenon be explained by the paracrine activity of these hormones in the pancreas rather than by their circulating levels? Indeed, SG has been shown to modify the cellular composition and activity of pancreatic islets, even increasing the residual pancreatic ghrelin cell-producing population (59). Furthermore, a ghrelin-positive effect on glucagon secretion has been reported using cultured mouse islets and alphaTC1 and InR1G9 alpha cell lines. However, ghrelin Knockout and Ghsr(-/-) Knockout mice exhibit no changes in plasma glucose patterns after OGTT or IPTT, suggesting a limited paracrine effect of ghrelin on pancreatic islets. It is possible that this paracrine effect also occurs in the case of glucagon secretion. Nonetheless, it is difficult to investigate these results in humans, and further efforts are needed.

Finally, SG has also been correlated to increases in the circulating levels of GIP in diabetic rodents (60). However, this relationship has not been confirmed in patients. The effect can be understood based on the idea that the anatomical rearrangements of the digestive tube due to SG surgery do not affect the duodenum. Other authors have reported increases in GIP in the immediate postoperative period. However, these levels normalized 12 months later (61). This change indicates a probable adaptive phenomenon in the digestive tube. Together, these data rule out the possible insulinotropic synergistic role of GIP as proposed in the case of RYGB.

## 6 Outlook, limitations and future prospects

In this review, we briefly discussed the physiology of glucagon, its implication in T2DM, and its role in glucose improvement after bariatric/metabolic surgery. Several insights have been gained from studies on the role of glucagon in improved glucose metabolism after metabolic and bariatric surgery, and one of the most important is that glucagon is not an anti-insulin hormone due to its reported insulinotropic actions and postsurgical high plasma levels, exhibiting a dual role in the regulation of glucose homeostasis. Another important aspect is the possible modulation of glucagon effects by other hormones whose expression is modified by surgery, such as GLP-1, ghrelin, or GIP. Many studies have been performed to elucidate these complex interactions, but many of them occur in the paracrine environment, and it is very difficult to translate this research from animal models to patients. Finally, metabolic/bariatric surgery has allowed the exploration of modifications and alternative sources of glucagon as parts of a compensatory mechanism of the anatomical and functional rearrangements derived from surgery.

However, many issues remain to be elucidated, which represent promising areas for future research. In this sense, the possible use of glucagon agonists to treat T2DM based on their insulinotropic effects is intriguing. Furthermore, the findings of the endocrine pancreas and functional assays performed in animals that have undergone metabolic/bariatric surgery support the study of glucagon and its phenomena of dumping and relapse in T2DM after surgery seems to be important. Thus, there remains significant areas to explore to understand the role of glucagon.

## Author contributions

The author's contributions were equal in the whole manuscript. All authors co-participate in references selection, reading, analysis and manuscript writing. The initial version of the manuscript was written by G-MP-A and J-AP-O. All authors contributed to the article and approved the submitted version.
